# Supplementary material for: Clinical feature and gene expression analysis in low prostate-specific antigen, high-grade prostate cancer
Source: PLoS One. 2025 Apr 15;20(4):e0321728. doi: 10.1371/journal.pone.0321728 (PMC11999122; doi:10.1371/journal.pone.0321728)
Supplement: Attachment — Submitted filename: Response to Reviewers.docx [file pone.0321728.s004.docx]

Reviewer #1: This study addresses a critical area in prostate cancer (PCa) research, specifically the clinical characteristics and gene expression patterns in patients presenting with low prostate-specific antigen (PSA) levels but high-grade disease. This subgroup is particularly concerning, as these patients often exhibit resistance to standard treatments and poor outcomes.
The study's large sample size enhances the reliability of its findings, and the comprehensive statistical methods employed (Kruskal-Wallis H, Pearson's chi-squared tests, and competing-risks regression) bolster the validity of the results. The focus on gene expression adds an important molecular dimension to the clinical observations, offering potential avenues for targeted therapeutic strategies. However, the author(s) should address the following points:
1. It's important to specify the type of this cohort study in the methods section if it is retrospective or prospective.
2. While the findings are significant, the study would benefit from additional exploration of the mechanisms driving the observed gene expression changes.
3. This study requires more explanation of the PI3K pathway, focusing on its role in prostate cancer.
4. The full terms of PI3K, AKT/mTOR, IGF-IGFR-PI3K-NFKB, and EGF-EGFR-PI3K-NFKB should be write as they first mentioned in the manuscript.

Overall, this research contributes valuable insights into the clinical features and molecular underpinnings of low PSA, high-grade prostate cancer. The identification of the PI3K signaling pathway as a potential therapeutic target warrants further investigation and may ultimately lead to improved management strategies for this challenging patient population.
Further research could elucidate how these alterations contribute to treatment resistance and could guide the development of targeted therapies.

Response to Reviewer #1

We sincerely appreciate your valuable feedback, which helped us identify several shortcomings in the paper. In response, we have made the necessary revisions and added relevant content accordingly.

1. We emphasized the use of the SEER database and the GEO database for conducting a retrospective study on Page 6 Line 74, and Page 8 Line 116, respectively.
2. We analyzed the similarities between gene sets and summarized several enriched pathways, including the polycomb repressive complex 2, epidermal growth factor receptor, retrograde axonal transport, tumor necrosis factor/nuclear factor-κB, Rho guanine nucleotide exchange factor/RhoA, and phosphoinositide 3-kinase signaling (PI3K) pathways. We then discussed the impact of these pathways on prostate cancer (from Page 19 Line 251 to Page 22 Line 298) and outlined potential therapeutic strategies targeting them (from Page 22 Line 312 to Page 23 Line 322).
3. We have added a discussion on the role of the PI3K pathway in prostate cancer, highlighting its promotion of prostate cancer cell survival by inhibiting apoptosis, advancing cell cycle progression, and increasing proliferation rates. Additionally, we highlighted PI3K/protein kinase B signaling drives epithelial-mesenchymal transition, contributing to metastasis (from Page 16 Line 206 to Page 17 Line 211).
4. Thank you for your helpful suggestion. We have included the full names of the abbreviations for each pathway upon their first mention in the manuscript.

Reviewer #2: This paper looked into the clinical characteristics and gene expression mechanisms in prostate cancer patients with low PSA levels.

Accessing the SEER database, the authors categorized data concerning 365,558 patients into four groups, according to PSA levels and Grade Group. The paper reports that, compared to other subtypes, patients with low PSA levels and high-grade disease exhibited higher tumor stage and elevated prostate cancer-specific mortality, possibly via the activation of PI3K signaling pathway. The authors conclude that screening and post-treatment monitoring should include physical examination, imaging findings, and other biomarkers.

This study emphasized the urgent need for novel biomarkers and modalities with high sensitivity and specificity to facilitate prognostication and the development of individualized treatment options and follow-up strategies for prostate cancer patients. The research questions explored here – if repeated in a number of robust studies – can be of significance for the treatment and management of prostate cancer.

With that in mind, this reviewer wish the authors the very best with their research!

Response to Reviewer #2

During the revision process, we also identified several pathways, in addition to the PI3K pathway, which may be associated with poor prognosis of prostate cancer cases with low PSA levels and high-grade tumors. We hope these findings will contribute to improving the prognosis for these patients.
