# Supplementary material for: Clinical feature and gene expression analysis in low prostate-specific antigen, high-grade prostate cancer
Source: PLoS One. 2025 Apr 15;20(4):e0321728. doi: 10.1371/journal.pone.0321728 (PMC11999122; doi:10.1371/journal.pone.0321728)
Supplement: Attachment — Submitted filename: Response_to_Reviewers_auresp_2.docx [file pone.0321728.s005.docx]

Reviewer`s comments:

1. You should improve discussion section including compare and contrast ideas and reinforcing why your results are so important.
2. Change genes names into itatics as well as latin words.

Response to reviewer

We greatly appreciate the reviewer’s insightful feedback, and we have revised the manuscript accordingly.

1. **Significance of the Study**: First, our study identifies that prostate cancer (PCa) patients with low PSA levels and high-grade tumors tend to present with higher tumor stages and a significantly increased risk of prostate cancer-specific mortality (PCSM). This underscores the need for heightened clinical attention for these patients. Second, prior studies have also highlighted that this PCa subtype exhibits resistance to androgen deprivation therapy (ADT). Therefore, improving the prognosis of these patients is of paramount importance. Our findings contribute valuable insights toward addressing this issue. Specifically, we identified several enriched signaling pathways in this patient population. Existing research has shown that targeting these pathways with therapeutic agents can yield varying degrees of efficacy in treating PCa. Consequently, future studies should investigate the potential of these targeted therapies in improving outcomes for patients with low-PSA, high-grade PCa. Both of these points reinforce the significance of our research.

**Modifications to the Discussion**: We have made appropriate revisions to the discussion section, condensing some content, expanding on others, and re-organizing the structure for clarity. First, compared to previous studies, our research included patients with regional lymph node metastasis and distant metastasis, providing a more comprehensive cohort. Second, we emphasize that patients with low PSA levels and high-grade PCa exhibit resistance to ADT, highlighting the need for alternative treatment strategies. Lastly, we discuss the enriched signaling pathways identified in our study and the potential therapeutic agents, suggesting that further research is required to validate their efficacy in low-PSA, high-grade PCa.

2. We have carefully revised the manuscript to address the reviewer’s suggestion. All gene names, including *PTEN*, and Latin terms have been italicized in accordance with the requested formatting guidelines.
