# Supplementary material for: Clinical feature and gene expression analysis in low prostate-specific antigen, high-grade prostate cancer
Source: PLoS One. 2025 Apr 15;20(4):e0321728. doi: 10.1371/journal.pone.0321728 (PMC11999122; doi:10.1371/journal.pone.0321728)
Supplement: Attachment — Submitted filename: Response_to_Reviewers_auresp_3.docx [file pone.0321728.s006.docx]

Reviewer`s comments:

1. In my opinion, the manuscript still needs changes such as, indicating why you use GG classification? This is not an extended way to name clinical classification in PCa. Why do not change for universal classification?
2. According to discussion, it is true that is improved, but including subheading in this part is not common and make the text less fluid, so I will suggest to connect comparing and contrasting all these sections.

Response to reviewer

We greatly appreciate the reviewer’s insightful feedback, and we have revised the manuscript accordingly.

1. We thank the reviewers for their astute observation regarding grading terminology. In response to your suggestion, we have replaced "Grade Group" with "Gleason score" throughout the manuscript. Additionally, we have made corresponding revisions to the relevant content and updated the data in the tables to reflect this change.
2. We are grateful for the reviewers` insightful critique of our pathway-focused approach. In our study, we identified several enriched signaling pathways in prostate cancer (PCa) patients with low PSA levels and high grades. Given that these patients may resist androgen deprivation therapy, we hypothesized that pharmacological agents targeting these pathways could potentially improve clinical outcomes. To substantiate this hypothesis, we systematically compiled and compared evidence-based data from clinical trials for drugs associated with each pathway.

Our analysis revealed that inhibitors targeting the PI3K/AKT and RhoA signaling pathways have shown substantial clinical promise in PCa treatment, supported by robust trial data. Conversely, therapeutic agents directed at the PRC2 and NF-κB pathways remain in earlier developmental stages, with limited clinical validation to date. These findings highlight prioritized directions for future clinical investigations.

During the revision process, we streamlined the functional descriptions of these pathways to enhance the manuscript’s readability and focus, ensuring that the core findings remain central to the narrative.
